# *XRCC1* and *XPD* DNA repair gene polymorphisms: A potential risk factor for glaucoma in the Pakistani population

**Published:** 2011-05-04

**Authors:** Sajeela Yousaf, Muhammad Imran Khan, Shazia Micheal, Farah Akhtar, Syeda Hafiza Benish Ali, Moeen Riaz, Mahmood Ali, Pramila Lall, Nadia Khalida Waheed, Anneke I. den Hollander, Asifa Ahmed, Raheel Qamar

**Affiliations:** 1 Department of Biosciences, COMSATS Institute of Information Technology, Islamabad, Pakistan; 2 Al-Shifa Eye Trust Hospital, Rawalpindi, Pakistan; 3 Christian Hospital Taxila, Pakistan; 4 Shifa International Hospital, Islamabad, Pakistan; 5 Shifa College of Medicine, Islamabad, Pakistan; 6 Department of Ophthalmology, Radboud University Nijmegen Medical Centre, Nijmegen, The Netherlands

## Abstract

**Purpose:**

The present study was designed to determine the association of polymorphisms of the DNA repair genes X-ray cross-complementing group 1 (*XRCC1*) (c.1316G>A [rs25487]) and xeroderma pigmentosum complementation group D (*XPD*) (c.2298A>C [rs13181]) with primary open-angle glaucoma (POAG) and primary closed-angle glaucoma (PCAG).

**Methods:**

In this prospective case-control study, polymerase chain reaction-restriction fragment length polymorphism analysis was used to study the association of *XRCC1* and *XPD* with 160 POAG patients, 163 PCAG patients, and 193 unaffected controls.

**Results:**

*XRCC1* rs25487 was found to be significantly associated specifically with male POAG patients (χ^2^=13.2 [p=0.001]), only for the dominant model (odds ratio [OR]=2.65 [95% confidence interval [CI]=1.44–4.85], p<0.005). In addition *XPD* rs13181 was also found to be associated with male POAG patients (χ^2^=12.1 [p<0.005]), for both dominant (OR=2.44 [95% CI=1.33–4.47], p<0.005) as well as recessive model (OR=3.62 [95% CI=1.45–9.01], p<0.01). Combined genotypes of both the genes revealed that the heterozygote AC/GA was significantly associated with the male POAG patients (z=3.00 [p<0.001]). The AA/GG genotype was present at a higher frequency in the male controls and the AA/GA in the female controls and could thus have a protective role in males and females, respectively.

**Conclusions:**

We postulate that defects in the DNA repair genes *XRCC1* and *XPD* may possibly be associated with the progression of POAG in male patients of Pakistani origin.

## Introduction

Glaucoma is a leading cause of irreversible blindness, characterized by degeneration of the retinal ganglion cells and optic nerve damage. Among the two common clinical types primary open angle glaucoma (POAG) is the most prevalent form, which is characterized by an obstruction of the aqueous humor pathway as a result of trabecular meshwork degeneration or decay. The other form primary close angle glaucoma (PCAG) is characterized by closure of angle between the iris and trabecular meshwork, mainly because of anatomic abnormalities. Although the etiology of glaucoma is not fully understood, there is strong evidence to suggest that it is caused by a combination of environmental and genetic factors [1].

Several studies support the involvement of oxidative damage to DNA as a common factor of glaucomatous neurodegeneration, which occurs in different subcellular compartments of the retinal ganglion cells [2-4]. Many DNA repair genes and their polymorphisms including the Xeroderma pigmentosum complementation group D (*XPD,* also known as Excision Repair Cross-complementation group 2 [ERCC2]), and the X-ray cross-complementing group 1 (*XRCC1*) have been reported to be associated with several different diseases including glaucoma, which have been shown to be dependent upon the genetics and ethnicity of the individuals or environmental factors [5].

*XRCC1* on chromosome 19q13.2 encodes a multi-domain protein that interacts with nicked DNA and is involved in single-strand breaks and base excision repair (BER) pathway in response to DNA damage caused by ionizing radiation, alkylating agents and oxidation. Different *XRCC1* mutations have been shown to disrupt the function of the protein by affecting its binding to the substrate or by introducing changes in the catalytic domain [6]. A single nucleotide polymorphism (SNP; c.1316G>A; p.Arg399Gln, rs25487), located near the breast cancer C-terminal domain (BRCT), is involved in cell cycle checkpoint functions that are initiated in response to DNA damage. This BRCT domain interacts with the poly-ADP-ribose polymerase binding domain of Poly (ADP-ribose) polymerase-1, which is an enzyme involved in the BER pathway [7]. Therefore any change in the structure of BRCT has the potential to lead to defects in the detection of the DNA damage and hence the activation of the BER pathway. Although no direct functional evidence is available that the variant c.1316A allele interrupts the interaction between BRCT and Poly (ADP-ribose) polymerase-1, but indirect functional analysis using biomarkers, such as chromosomal aberrations, have shown that cancer patients with the c.1316A allele have a significantly higher number of chromosomal aberrations (such as deletions) and greater number of DNA adducts as compared to individuals with wild type c.1316G allele, indicative of defects in the BER pathway [8,9].

*XPD* encodes an ATP-dependent DNA helicase present 1.8 Mb downstream of *XRCC1* on chromosome 19q13.3, it is an important component of the Transcription Factor IIH that is involved in nucleotide excision repair (NER) of UV induced damage and removal of bulky adducts [5]. Genetic variations in *XPD* are associated with defects in the NER mechanism [10] resulting in autosomal recessive DNA repair disorders. Two SNPs in *XPD,* p.Asp312Asn (rs1799793) and p.Lys751Gln (rs13181), have been shown to be involved in susceptibility to various types of cancer in addition to various inherited and age related diseases [7,11]. The rs13181 affects an ATP-binding site of XPD and destroys its helicase activity, which is important for NER, but does not affect its transcriptional activity [12]. The lysine at codon 751 is assumed to be involved in interactions with the substrate of XPD, thus any substitution at this residue may produce changes in its function which can impair the DNA repair capacity [13].

Up to the present date there is no data available that shows a significant association of the polymorphisms of DNA repair genes with glaucoma in any specific ethnic group [14]. The aim of the present study was therefore to investigate the relationship between polymorphisms of the DNA repair genes and two different forms of glaucoma in the Punjabi Pakistani population. Our study is the first from Pakistan to report ethnic-based associations of the *XRCC1* and *XPD* polymorphisms with male POAG patients.

## Methods

### Patient selection criteria

This study conforms to the tenants of the Helsinki declaration and has been approved by the Departmental Review and Ethics Committee. All subjects were briefed about the study in their local language and informed written consent was obtained from them before obtaining their blood samples. The study group consisted of Punjabi’s which are the major ethnic group of Pakistan. The affected arm was composed of 323 Punjabi patients consisting of 160 POAG (80 males, 80 females) and 163 PCAG cases (81 males, 82 females), in addition DNA samples of 193 unaffected (101 males, 92 females) age and sex matched Punjabi control individuals were also studied. The inclusion criteria for patients/controls, clinical examination, as well as collection and processing of the whole blood have been described previously [15]. In addition to the isolated glaucoma patients, 9 Punjabi families (29 patients, 68 unaffected) of congenital POAG were also screened to study the association of the two SNPs in these families. Briefly, blood samples from controls, patients and family members were collected in EDTA tubes and genomic DNA was extracted by a conventional phenol chloroform method [16].

### Polymerase chain reaction (PCR) amplification of genomic DNA

The *XRCC1* codon 399 and *XPD* codon 751 polymorphisms were detected by PCR-RFLP. A 242 base pair (bp) fragment containing the polymorphism rs25487 (c.1316G>A; p.Arg399Gln) in exon 10 of the *XRCC1* gene was amplified using the primers: 5′-CCC CAA GTA CAG CCA GGT C-3′ (forward) and 5′-TGT CCC GCT CCT CTC AGT AG-3′ (reverse). A 436-bp fragment containing the polymorphism rs13181 (c.2298A>C; p.Lys751Gln) in exon 23 of the *XPD* gene was amplified using the primers 5′-GCC CGC TCT GGA TTA TAC G-3′(forward) and 5′-CTA TCA TCT CCT GGC CCC C-3′ (reverse). The 25 µl-PCR reaction for each polymorphism contained 1× PCR buffer, 1.5 mM MgCl_2_ (Fermentas, Burlington, Ontario), 10 pmol of each primer, 0.2 mM deoxy nucleotide triphosphates (dNTPs; Fermentas, Burlington, Ontario), 1 U Taq polymerase (Biotools, Madrid, Spain) and 50 ng genomic DNA as template. The PCR thermal cycling profile consisted of an initial denaturation at 95 °C for 5 min followed by 35 cycles of 1 min DNA denaturation at 95 °C, primer annealing for 45 s at 60 °C, 1 min extension at 72 °C, followed by one cycle of a final extension step for 7 min at 72 °C. The amplifications were performed with an Applied BioSystem Gene Amp^®^ PCR system 2700 (Foster City, CA).

### Restriction fragment length polymorphism (RFLP) analysis of *XRCC1*

**Figure 1:**
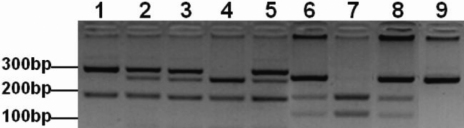
Agarose gel electrophoresis of the polymerase chain reaction based restriction fragment length polymorphism (PCR-RFLP) analysis of *XPD* and *XRCC1*. (Lanes 1–5) PstI digested fragments of exon 23 c.2298A>C *XPD* SNP. (Lane 1) AA genotype consisting of two fragments; 290 and 146 bp. (Lanes 2, 3, and 5) AC genotype consisting of four fragments; 290, 227, 146 and 63 bp. (Lane 4) CC genotype consisting of three fragments; 227, 146, and 63 bp. (Lanes 6–9) MspI digested fragments of exon 10 c.1316G>A *XRCC1* SNP. (Lane 7) GG genotype consisting of two fragments; 148 and 94 bp. (Lanes 6 and 8) AG genotype consisting of three fragments; 242, 148, and 94 bp. (Lane 9) AA genotype consisting of an undigested fragment of 242 bp.

The PCR products were digested with MspI restriction enzyme (Fermentas, Burlington, Ontario) at 37 °C overnight, followed by electrophoretic separation on 2.5% agarose gels, along with a 100 bp DNA ladder (Invitrogen Inc., Carlsbad, CA). The “A” allele of the polymorphism rs25487 degenerates a MspI restriction site, resulting in two fragments of 148 and 94 bp for the GG genotype, three fragments of 242, 148, and 94 bp of the AG genotype, and a single fragment of 242 bp for the AA genotype (Figure 1).

### RFLP analysis of *XPD*

The PCR amplified products were digested with PstI restriction enzyme (Fermentas) overnight at 37 °C and the fragments were resolved on 4% agarose gel. The “C” allele of the polymorphism rs13181 creates a PstI restriction site. The AA genotype results in two fragments of 290 and 146 bp, four fragments of 290, 227, 146, and 63 bp for the AC genotype, and three fragments of 227, 146, and 63 bp for the CC genotype (Figure 1).

### Sequence analysis of *XRCC1* and *XPD* genotypes

**Figure 2:**
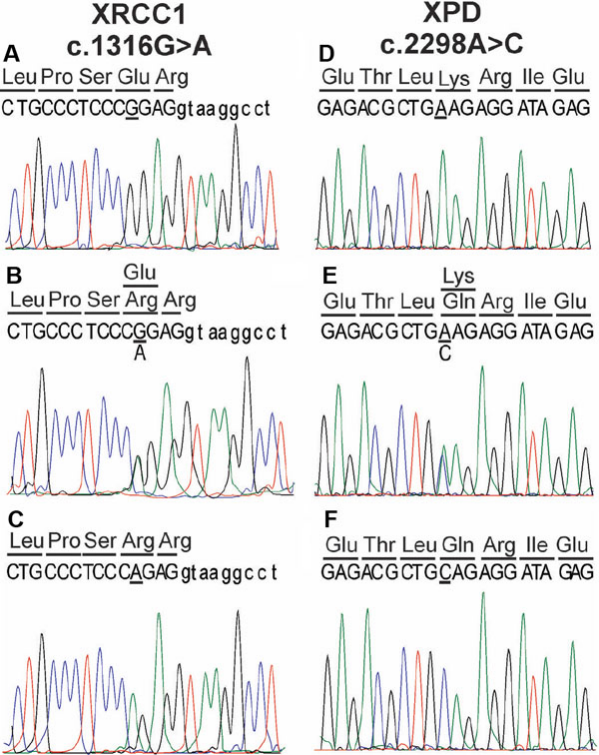
Sequence chromatograms of the region of *XRCC1* and *XPD* containing the respective SNP’s. **A**-**C**: *XRCC1* c.1316G>A (p.Arg399Gln) variants, homozygous GG (**A**), heterozygous GA (**B**), and homozygous AA (**C**). **D**-**F**: *XPD* c.2298A>C (p.Lys751Gln) variants, homozygous AA (**D**), heterozygous AC (**E**), and homozygous CC (**F**).

To validate the RFLP results 5 random samples of each genotype of rs25487 (n=15) and rs13181 (n=15) were sequenced using the primers described above for the PCR amplification of these SNP’s (Figure 2A-F).

### Statistical analysis

The demographic features (age, Intra Ocular Pressure [IOP], and Cup to Disk Ratio [CDR]), genotype, and allele distribution of both patient and controls were analyzed by using SPSS (ver. 16; SPSS, Chicago, Il) and the level of significance of the clinical parameters were tested using Minitab (ver. 15; Minitab, Chicago, Il). The genotype and allele frequency differences between POAG, PCAG, controls, and affected/non-affected family members were compared by chi square (χ^2^) and/or z tests, a p-value <0.05 was considered to be statistically significant.

## Results

In the present study the mean age of controls was 39.7±11.9 and 41.3±13.7 for POAG, and 43.6±15.8 years in case of PCAG patients, there being no statistically significant difference in the ages of the three groups. In most of the patients the disease was diagnosed at an advance stage, therefore an early onset of the disease cannot be precluded. In all the patients there was no evidence of systemic diseases like diabetes, hypertension, etc. in addition the development of secondary glaucoma due to some other eye anomaly was also ruled out. The mean IOP in the controls was 13.70±1.86 mmHg, which was significantly higher in the POAG and PCAG patients (26.00±9.27 mmHg and 26.50±10.80 mmHg, respectively, p<0.001). Similarly, the CDR in the control group was 0.30±0.11, which was significantly larger in the POAG and PCAG patients (0.83±.30 and 0.65±.23, respectively, p<0.001).

### *XRCC1* genotyping

For the POAG patients (Male+Female) there was no significant difference in the overall genotype (GG, GA, and AA) distribution as compared to Male+Female control group (χ^2^=5.69 [p>0.05]; Table 1), although the frequency of the GA genotype was significantly higher in the patients (z=2.29 [p<0.05]). However, when the data were analyzed along the lines of gender a highly significant difference was observed between the genotype distribution of the male POAG and control samples (χ^2^=13.2 [p=0.001]; Table 1), while no significant difference was observed in the case of the females (χ^2^=2.93 [p>0.05]; Table 1). The difference in the genotype frequency of the male POAG patients was significant only under a dominant model (odds ratio [OR]=2.65 [95% confidence interval [CI]=1.44–4.85], p<0.005). As opposed to this analysis of the PCAG samples revealed no significant difference in the overall genotype distribution of Male+Female as compared to the controls (χ^2^=0.25 [p>0.05]), as well as when the samples were distributed along the lines of gender (Table 1).

**Table 1:** Genotype and allele frequency comparison of *XRCC1* (c.1316G>A [rs25487]) in controls and patients and with gender distribution.

**Genotype**	**Male+Female Controls (n=193)**	**Male+Female POAG (n=160)**	**p (z)**	**p (χ^2^)**	**OR (95% CI)**	**Male+Female PCAG (n=163)**	**p (z)**	**p (χ^2^)**	**OR (95% CI)**
GG	98(51%)	70(44%)	>0.05(1.32)	>0.05 (5.69)	DM=1.33(0.87–2.02) p>0.05 RM=0.65(0.35–1.21) p>0.05	79(48%)	>0.05(0.43)	>0.05(0.25)	DM=1.1(0.72–1.66) p>0.05 RM=1.13(0.64–1.97) p>0.05
GA	65(34%)	73(46%)	***<0.05(2.29)***			56(34%)	>0.05(0.13)		
AA	30(15%)	17(10%)	>0.05 (1.35)			28(17%)	>0.05(0.42)		
**Allele**	**Male+Female Controls**	**Male+Female POAG**	**p (χ^2^)**	**OR (95% CI)**	**Male+Female PCAG**	**p (χ^2^)**	**OR (95% CI)**		
G	261(68%)	213(67%)	>0.05(0.09)	1.05(0.77–1.44) p>0.05	214(66%)	>0.05(0.31)	1.09(0.8–1.49)		
A	125(32%)	107(33%)			112(34%)		p>0.05		
**Genotype**	**Male Controls (n=101)**	**Male POAG (n=80)**	**p (z)**	**p (χ^2^)**	**OR (95% CI)**	**Male PCAG (n=81)**	**p (z)**	**p (χ^2^)**	**OR (95% CI)**
GG	58(57%)	27(34%)	***<0.005(3.17)***	***0.001(13.2)***	DM=2.65(1.44–4.85) ***p<0.005*** RM=0.82(0.35–1.91) p>0.05	38(47%)	>0.05(1.41)	>0.05(2.05)	DM=1.53(0.85–2.74) p>0.05 RM=1.2(0.55–2.62) p>0.05
GA	28(28%)	43(54%)	***<0.001(3.56)***			29(36%)	>0.05(1.17)		
AA	15(15%)	10(12%)	>0.05(0.46)			14(17%)	>0.05(0.45)		
**Allele**	**Male Controls**	**Male POAG**	**p (χ^2^)**	**OR (95% CI)**	**Male PCAG**	**p (χ^2^)**	**OR (95% CI)**		
G	144(71%)	97(61%)	***<0.05(4.56)***	1.61(1.04–2.5) ***p<0.05***	105(65%)	>0.05(1.33)	1.3(0.83–2.03) p>0.05		
A	58(29%)	63(39%)			55(35%)				
**Genotype**	**Female Controls (n=92)**	**Female POAG (n=80)**	**p (z)**	**p (χ^2^)**	**OR (95% CI)**	**Female PCAG (n=82)**	**p (z)**	**p (χ^2^)**	**OR (95% CI)**
GG	40(44%)	43(54%)	>0.05(1.34)	>0.05(2.93)	DM=0.66(0.36–1.21) p>0.05 RM=0.49(0.2–1.25) p>0.05	41(50%)	>0.05(0.86)	>0.05(1.04)	DM=0.77(0.42–1.4) p>0.05 RM=1.06(0.48–2.32) p>0.05
GA	37(40%)	30(37%)	>0.05(0.36)			27(33%)	>0.05(1.0)		
AA	15(16%)	7(9%)	>0.05(1.48)			14(17%)	>0.05(0.14)		
**Allele**	**Female Controls**	**Female POAG**	**p (χ^2^)**	**OR (95% CI)**	**Female PCAG**	**p (χ^2^)**	**OR (95% CI)**		
G	117(64%)	116(72%)	>0.05(3.11)	0.66(0.42–1.05) p>0.05	109(66%)	>0.05(0.32)	0.88(0.57–1.37) p>0.05		
A	67(36%)	44(28%)			55(34%)				

DM=dominant model, RM=recessive model. Significant p values (<0.05) are in bold italics.

The frequency of the risk allele “A” was 39% in the male POAG patients, which was significantly higher from the 29% in the male controls (χ^2^=4.56 [p<0.05], OR=1.61 [95% CI=1.04–2.5], p<0.05; Table 1).

### *XPD* genotyping

The overall genotype (AA, AC, and CC) distribution in the POAG (Male+Female) and controls was significantly different from each other (χ^2^=8.75 [p=0.01]; Table 2), which was statistically significant under the dominant model only (OR=1.89 [95% CI=1.23–2.92], p=0.005). As opposed to this no significant difference between the genotype frequencies of PCAG and control subjects was found (χ^2^=5.68 [p>0.05]).

**Table 2:** Genotype and allele frequency comparison of *XPD* (c.2298A>C [rs13181]) in controls and patients and with gender distribution.

**Genotype**	**Male+Female Controls (n=193)**	**Male+Female POAG (n=160)**	**p (z)**	**p (χ^2^)**	**OR (95% CI)**	**Male+Female PCAG (n=163)**	**p (z)**	**p (χ^2^)**	**OR (95% CI)**
AA	92(48%)	52(33%)	***<0.005(2.89)***	0.01(8.75)	DM=1.89(1.23–2.92) ***p=0.005*** RM=1.62(0.91–2.86) p>0.05	58(36%)	***<0.05(2.3)***	>0.05 (5.68)	DM=1.65(1.08–2.53) ***p<0.05*** RM=1.52(0.86–2.69) p>0.05
AC	76(39%)	77(48%)	>0.05(1.65)			75(46%)	>0.05(1.26)		
CC	25(13%)	31(19%)	>0.05(1.64)			30(18%)	>0.05(1.42)		
**Allele**	**Male+Female Controls**	**Male+Female POAG**	**p (χ^2^)**	**OR (95% CI)**	**Male+Female PCAG**	**p (χ^2^)**	**OR (95% CI)**		
A	260(55%)	181(57%)	***<0.005(8.7)***	1.59(1.17–2.15) ***p<0.005***	191(59%)		1.46(1.07–1.98) ***p<0.05***		
C	126(33%)	139(43%)			135(41%)	***<0.05(5.85)***			
**Genotype**	**Male Controls (n=101)**	**Male POAG (n=80)**	**p (z)**	**p (χ^2^)**	**OR (95% CI)**	**Male PCAG (n=81)**	**p (z)**	**p (χ^2^)**	**OR (95% CI)**
AA	56(55%)	27(34%)	***<0.005(2.91)***	***<0.005(12.1)***	DM=2.44(1.33–4.47) ***p<0.005*** RM=3.62(1.45–9.01) ***p<0.01***	33(41%)	***0.05(1.97)***	>0.05 (4.71)	DM=1.81(1.00–3.26) ***p=0.05*** RM=2.1(0.8–5.55) p>0.05
AC	38(38%)	36(45%)	>0.05(1.00)			37(46%)	>0.05(1.1)		
CC	7(7%)	17(21%)	***0.005(2.82)***			11(13%)	>0.05(1.49)		
**Allele**	**Male Controls**	**Male POAG**	**p (χ^2^)**	**OR (95% CI)**	**Male PCAG**	**p (χ^2^)**	**OR (95% CI)**		
A	150(74%)	90(56%)	***<0.001(12.96)***	2.24(1.44–3.49) ***p<0.001***	103(64%)	***<0.05(4.84)***	1.65(1.06–2.6) ***p<0.05***		
C	52(26%)	70(44%)			59(36%)				
**Genotype**	**Female Controls (n=92)**	**Female POAG (n=80)**	**p (z)**	**p (χ^2^)**	**OR (95% CI)**	**Female PCAG (n=82)**	**p (z)**	**p (χ^2^)**	**OR (95% CI)**
AA	36(39%)	25(31%)	>0.05(1.08)	>0.05(1.77)	DM=1.41(0.76–2.65) p>0.05 RM=0.87(0.41–1.87) p>0.05	25(31%)	>0.05(1.19)	>0.05 (1.44)	DM=1.47(0.78–2.74) p>0.05 RM=1.2(0.6–2.55) p>0.05
AC	38(41%)	41(51%)	>0.05(1.31)			38(46%)	>0.05(0.67)		
CC	18(20%)	14(18%)	>0.05(0.35)			19(23%)	>0.05(0.58)		
**Allele**	**Female Controls**	**Female POAG**	**p (χ^2^)**	**OR (95% CI)**	**Female PCAG**	**p (χ^2^)**	**OR (95% CI)**		
A	110(60%)	91(57%)	>0.05(0.3)	1.13(0.73–1.73) p>0.05	88(54%)	>0.05(1.33)	1.3(0.84–1.96) p>0.05		
C	74(40%)	69(43%)			76(46%)				

DM=dominant model, RM=recessive model. Significant p values (<0.05) are in bold italics.

When the POAG data were split along the lines of gender, only the male genotype distribution was found to be significantly different from the controls (χ^2^=12.1 [p<0.005]), which was significant under the dominant model (OR=2.44 [95% CI=1.33–4.47], p<0.005) as well as the recessive model (OR=3.62 [95% CI=1.45–9.01], p<0.01).

Compared to the male control individuals (26%) the frequency of the risk allele “C” was significantly higher in the POAG patients (44%) (χ^2^=12.96 [p<0.001], OR=2.24 [95% CI=1.44–3.49], p<0.001).

### Combined Genotypes of *XRCC1* and *XPD* genes

The combined genotypes of *XPD* and *XRCC1* of the controls (Male+Female) was significantly different in case of POAG (χ^2^=20.44 [p=0.01]) and also in the case of male POAG patients (χ^2^=27.04 [p<0.001]), but not for female POAG patients (χ^2^=13.01 [p>0.05]) or for any distribution in the case of PCAG. In addition the combined genotype, AC/GA of the POAG patients (Male+Female) when compared to the controls was found to be present at a significantly higher frequency (z=3.42 [p<0.001], which was solely because of the significantly higher frequency of AC/GA in the male patients (z=3.00 [p<0.001]) and not because of the female patients (z=1.82 [p>0.05]; Table 3), though the female patients AC/GA frequency was greater than the female controls (24% versus 13%) but not at a statistically significant level. The combined genotypes AA/GG, was present at a higher frequency in the controls (Male+Female) as compared to POAG patients (Male+Female; z=2.14, [p<0.05]), which was solely because of the higher frequency of this combined genotype in the male controls (z=3.12 [p<0.001]). While the AA/GA combined genotype was present at a higher frequency in the female controls as compared to female POAG and PCAG samples (z=2.17 [p<0.05] and z=2.24 [p<0.05], respectively).

**Table 3:** *XPD* and *XRCC1* combined genotype comparison of controls and patients.

***XPD*/ *XRCC***	**Male+Female Controls (n=193)**	**Male+Female POAG (n=160)**	**p (χ^2^)**	**p (z)**	**Male+Female PCAG (n=163)**	**p (χ^2^)**	**p (z)**
AA/GG	48(25%)	25(15%)	***0.01 (20.44)***	***<0.05(2.14)***	31(19%)	>0.05 (7.18)	>0.05(1.32)
AA/GA	30(16%)	19(12%)		>0.05(0.99)	18(11%)		>0.05(1.24)
AA/AA	14(7%)	8(5%)		>0.05(0.87)	9(5%)		>0.05(0.66)
AC/GG	40(21%)	29(18%)		>0.05(0.61)	35(21%)		>0.05(1.72)
AC/GA	26(13%)	45(28%)		***<0.001(3.42)***	26(16%)		>0.05(0.66)
AC/AA	10(5%)	3(2%)		>0.05(1.64)	14(9%)		>0.05(1.28)
CC/GG	10(5%)	16(10%)		>0.05(1.73)	13(8%)		>0.05(1.07)
CC/GA	9(4%)	9(6%)		>0.05(0.41)	12(7%)		>0.05(1.08)
CC/AA	6(3%)	6(4%)		>0.05(0.33)	5(3%)		>0.05(0.02)
***XPD*/ *XRCC***	**Male Controls (n=101)**	**Male POAG (n=80)**	**p (χ^2^)**	**p (z)**	**Male PCAG (n=81)**	**p (χ^2^)**	**p (z)**
AA/GG	36(35%)	12(15%)	***<0.001 (27.04)***	***<0.001(3.12)***	17(21%)	>0.05 (9.60)	<0.05(2.16)
AA/GA	11(11%)	12(15%)		>0.05(0.82)	11(14%)		>0.05(0.55)
AA/AA	9(9%)	3(4%)		>0.05(1.39)	5(6%)		>0.05(0.69)
AC/GG	20(20%)	9(11%)		>0.05(1.56)	17(21%)		>0.05(0.20)
AC/GA	14(14%)	26(33%)		***<0.001(3.00)***	12(15%)		>0.05(0.18)
AC/AA	4(4%)	1(1%)		>0.05(1.10)	8(10%)		>0.05(1.60)
CC/GG	2(2%)	6(7%)		>0.05(1.79)	4(5%)		>0.05(1.11)
CC/GA	3(3%)	5(6%)		>0.05(1.07)	6(7%)		>0.05(1.37)
CC/AA	2(2%)	6(7%)		>0.05(1.79)	1(1%)		>0.05(0.39)
***XPD*/ *XRCC***	**Female Controls (n=92)**	**Female POAG (n=80)**	**p (χ^2^)**	**p (z)**	**Female PCAG (n=82)**	**p (χ^2^)**	**p (z)**
AA/GG	12(13%)	13(16%)	>0.05 (13.01)	>0.05(0.60)	14(17%)	>0.05 (5.57)	>0.05(0.74)
AA/GA	19(21%)	7(9%)		***<0.05(2.17)***	7(8%)		***<0.05(2.24)***
AA/AA	5(5%)	5(6%)		>0.05(0.23)	4(5%)		>0.05(0.17)
AC/GG	20(22%)	20(25%)		>0.05(0.50)	18(22%)		>0.05(0.03)
AC/GA	12(13%)	19(24%)		>0.05(1.82)	14(17%)		>0.05(0.74)
AC/AA	6(6%)	2(3%)		>0.05(1.25)	6(7%)		>0.05(0.21)
CC/GG	8(9%)	10(12%)		>0.05(0.81)	9(11%)		>0.05(0.51)
CC/GA	6(6%)	4(5%)		>0.05(0.43)	6(7%)		>0.05(0.21)
CC/AA	4(4%)	0(0%)		>0.05(1.89)	4(5%)		>0.05(0.17)

Significant p values (<0.05) are in bold italics.

**Figure 3:**
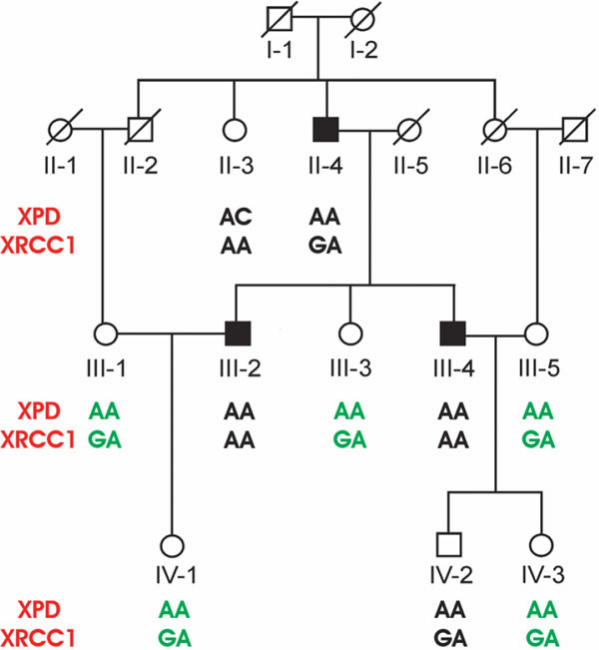
Pedigree of the congenital primary open angle glaucoma (POAG) family. Normal females are represented by circles and males by squares. Filled squares represent affected males. Deceased individuals are designated with a slanting line across the symbol. *XPD* and *XRCC1* genotypes are shown for each genotyped individual and the protective genotype (AA/GA) in the females is indicated with green letters.

In addition to the isolated glaucoma samples the genotype, allele and combined genotype data were also obtained of 9 POAG Punjabi families consisting of 29 affected (19 male, 10 female) and 68 non-affected (33 male, 35 female). As in the sporadic cases the combined genotype AC/GA was found at a higher frequency in the males from the affected families (26%) as compared to the random control population (14%) but this difference was statistically not significant (z=1.3 [p>0.05]; data not shown). As opposed to this the combined genotype AA/GG was not found in any of the affected male in the families, which when compared with the healthy control males 35% distribution was statistically significant (z=3.11 [p=0.001]). The AA/GA genotype was also present at a higher frequency in the female control population (21%) as compared to the female patients in the families (10%), though this was not statistically significant (z=0.8 [p>0.05]), this genotype was present at a particularly higher frequency in the non-affected females of one family (Figure 3).

Of the 15 XRCC1 and 15 XPD samples that were sequenced all of the sequence data matched with the RFLP results thus validating the latter data (Figure 2).

## Discussion

The integrity of the genome is continuously challenged exogenously and endogenously by DNA damaging agents, which induce a wide variety of lesions in the DNA including single and double strand breaks, oxidative lesions and pyrimidine dimer formation [4]. These lesions are efficiently repaired by several pathways that include BER and NER mechanisms, which are regulated by DNA repair genes [17-19]. Of these, BER is believed to be the major pathway for repairing deaminated bases and bases with oxidative damage caused by reactive oxygen species, BER can also be involved in repairing alkylated bases [20]. As opposed to this the NER pathway is involved in removing short segments of nucleotides containing damaged bases [21].

XRCC1, a component of the BER cascade acts as a scaffold for other enzymes e.g., DNA ligase, DNA polymerase, and poly ADP-ribose polymerase (a zinc finger containing enzyme that detects DNA strand breaks) [22]. Due to its central role in the BER pathway, amino acid changes in XRCC1 can have detrimental effects on its activity and subsequently on the BER mechanism. Similarly XPD has also been shown to play a vital role in the NER pathway and thus any non-synonymous amino acid changes in XPD can potentially result in a defective NER mechanism [10].

Unal et al. [23] reported *XRCC1* and *XPD* polymorphisms to be associated with cataract. In diseases such as glaucoma, oxidative stress (which could be caused by raised IOP in the eye) has been shown to increase damage to the DNA and other macromolecules [23]. Elevated IOP is the prime characteristic of PCAG [24] whereas degenerative changes in the trabecular meshwork due to oxidative DNA damage is a major contributing factor in the progression of POAG [2]. If the oxidative stress induced DNA damage in PCAG and POAG remains unchecked, due to failure of the repair mechanism, it would result in enhanced DNA damage which might lead to neurodegeneration of the optic nerve [3,25,26].

Au et al. [27] have previously reported that due to DNA repair defects there was an increase in the risk of chromosomal aberrations in individuals who were heterozygous or homozygous for the derived alleles of p.Arg399Gln and p.Lys751Gln, this phenomenon was also observed when the combined genotypes of these individuals were studied. To date there is no documented evidence that shows an association of the *XRCC1* polymorphism rs25487 with glaucoma [14]. In the current study the association of the *XRCC1* polymorphism with only the male POAG patients can probably be attributed to the significantly higher proportion of the GA genotype in patients as compared to controls. We therefore postulate that in our study population *XRCC1* rs25487 has a gender specific association only with male POAG patients. We propose that although the *XRCC1* polymorphisms might not directly participate in causing glaucoma but it can act as a hypomorph and thus could be involved in accelerating the progression of the DNA damage in the latter stages of the disease by hindering the DNA repair mechanism in male patients. This is in agreement with the observation that oxidative stress affects the optic nerve [25,26], which could possibly be via a stress induced DNA damage mechanism where the damage is not corrected timely due to a defective BER pathway.

Guven et al. [14] did not observe an association of *XRCC1* or *XPD* polymorphisms with glaucoma in the Turkish population, which was attributed to small sample size and lack of ethnic homogeneity by the authors. In addition till date all the association studies of *XRCC1* or *XPD* polymorphisms with glaucoma have not analyzed the data along the lines of gender or ethnic background. We have not only studied an ethnically pure Punjabi population, which comprises roughly 60% of the total Pakistani population but have also analyzed the data along the lines of gender and found an association of *XRCC1* rs25487 and *XPD* rs13181 with male POAG patients. One of the possible reasons for this gender specific association could lie in the life style of the Punjabi’s, where 80% of the population are farmers and live in rural areas. Being an underdeveloped country antiquated methods of farming are still in practice in Punjab where mainly the males do most of the labor intensive outdoor work, this results in a higher degree of exposure of the males to ambient sunlight and temperature. Such exposures have been shown to be one of the etiological factors causing age related ocular diseases like cataract, pterygium and macular degeneration [27]. We thus believe that the higher susceptibility of the Punjabi males toward the development of the disease, as compared to the females who mostly remain indoors, is because of the higher exposure of the males to UV induced damage from the sunlight.

Functional analysis conducted by Lunn et al. [28] provides an insight into the potential association of these SNPs with disease and hence supports our results, the authors demonstrated that sequence alterations, particularly at codon 751 of the *XPD,* may alter the protein product resulting in suboptimal repair of X-ray induced DNA damage. Moreover it has also been reported by Lehmann et al. [10] that mutations in *XPD* disrupt its enzymatic functions and cause defects in NER. We therefore hypothesize that the association of the *XPD* polymorphism with POAG could be due to a defective NER pathway that failed to repair the DNA damage caused by oxidative stress.

The combined genotype data revealed a significant association of the different combinations with POAG as well as PCAG but in a gender specific manner. The association of the AC/GA combined heterozygotes with male POAG patients is probably because of the presence of a higher number of *XRCC1* heterozygotes (i.e., G/A) in POAG and not because of the AC heterozygotes of *XPD*. This combined genotype is also found to be present at a higher frequency in the affected males from the families, though the difference is statistically non-significant but that is probably due to the smaller sample size of the affected males in the families.

Au et al. [9], in their functional characterization of *XRCC1* and *XPD* SNP’s showed that the variant genotype of the *XRCC1* might only interact with variant alleles of the other factors of BER pathways, but not with the variant genotype of *XPD* that belongs to the NER pathway.

They also showed that the ancestral *XRCC1* (A) and *XPD* (G) as well as the ancestral combined genotypes (AA/GG) were protective in nature. In our study this association seems to be a male specific phenomenon as the ancestral combined genotype (AA/GG) is present at a much lower frequency in the female controls as compared to the male controls. In the latter group this combined genotype seems to have a protective role against the development of glaucoma as it is present at a higher frequency in the controls as compared to the patients. This combined genotype also seems to play a role in providing protection to the males in the families as none of the patients had this combined genotype. Whereas in the case of sporadic female patients the combined genotype AA/GA seems to play a protective role, which is also the case in females from the families, particularly in one family in which this genotype was exclusively present in the non-affected females (Figure 3).

To validate these results in other populations, it must be kept in mind that any disease susceptibility or protective role these combined genotypes play could only be cross comparable in different populations if similarly elevated genotype frequencies are seen in the glaucoma patients in those populations as compared to normal control individuals.

In summary, our study is the first report that shows an association between the *XRCC1* rs25487 and *XPD* rs13181 polymorphisms in Pakistani male primary glaucoma patients belonging to the dominant ethnic Punjabi group. We propose that defects in the BER and NER pathway are probably associated with POAG in a gender and ethnic group specific manner. In addition we find that the AC/GA combined genotypes might be associated with increased risk for chromosomal damage that could be due to the reduced DNA repair functions or selective population association of these polymorphisms with the disease in the Pakistani Punjabi male patients and the AA/GG genotype seems to have a protective role against POAG in Punjabi male patients, while the AA/GA genotype is protective in Punjabi female POAG and PCAG patients.
